# Characterization of a Thermostable α-Amylase from *Bacillus licheniformis* 104.K for Industrial Applications

**DOI:** 10.3390/microorganisms13081757

**Published:** 2025-07-28

**Authors:** Askar Kholikov, Khushnut Vokhidov, Azizjon Murtozoyev, Zoé S. Tóth, Gergely N. Nagy, Beáta G. Vértessy, Akhmadzhan Makhsumkhanov

**Affiliations:** 1 Institute of Microbiology, Uzbekistan Academy of Sciences, Tashkent 100047, Uzbekistan; asqarxoliqov01@gmail.com (A.K.); vohidov.x@gmail.com (K.V.); murtozoyevazizjon@gmail.com (A.M.); 2 Department of Applied Biotechnology and Food Science, Faculty of Chemical Technology and Biotechnology, Budapest University of Technology and Economics, 1111 Budapest, Hungary; nagy.gergely.nandor@vbk.bme.hu; 3 Institute of Molecular Life Sciences, HUN-REN Research Centre for Natural Sciences, 1117 Budapest, Hungary; toth.zoe@ttk.hu; 4 Doctoral School of Biology, Institute of Biology, ELTE Eötvös Loránd University, 1117 Budapest, Hungary

**Keywords:** *Bacillus licheniformis*, α-amylase, GH13_5 subfamily, thermostability, recombinant enzyme, affinity chromatography

## Abstract

This study describes the characterization of a novel thermostable α-amylase from a *Bacillus licheniformis* 104.K strain isolated from the Kashkadarya region of Uzbekistan. Phylogenetic analysis revealed that the thermostable α-amylase belongs to glycoside hydrolase family 13 subfamily 5 (GH13_5) and shares high sequence similarity with known α-amylases. Our results demonstrate that the recombinant α-amylase exhibits optimal activity at pH 6.0 and 90 °C, retaining full activity after 30 min at 60 °C. The addition of CaCl_2_ significantly enhanced thermostability, with the enzyme retaining more than 95% of its initial activity at 70 °C after 30 min. Our findings indicate that α-amylase from *B. licheniformis* 104.K is a functional, thermostable enzyme with potential industrial applications. This study highlights the commercial significance of thermostable amylases and the need to identify novel, cost-effective, and sustainable sources. The results of this study will contribute to the fields of enzyme applications, stabilizing additives, and genetic engineering of thermostable genes.

## 1. Introduction

**Figure 1:**
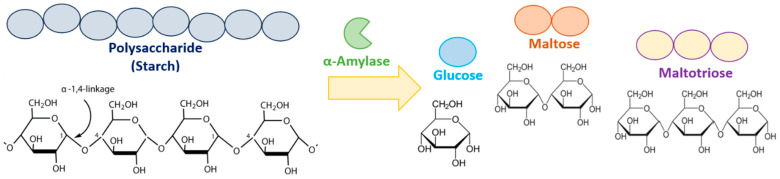
Mechanism of starch hydrolysis by α-amylase. The enzyme cleaves internal α-1,4-glycosidic linkages in polysaccharide chains, producing glucose, maltose, and maltotriose.

α-Amylases (E.C. 3.2.1.1) are essential industrial enzymes primarily utilized in starch processing. They hydrolyze α-1,4-glycosidic bonds of starch, releasing mono- and oligosaccharide products, including glucose, maltose, and maltotriose (Figure 1) [1,2,3]. According to the Carbohydrate-Active enzymes (CAZy) database classification, α-amylases belong to the glycoside hydrolase (GH) families, such as GH13, GH57, GH119, and GH126 [4,5]. GH13 is the largest family of amylolytic enzymes and is divided into 47 subfamilies [6].

α-Amylase ranks first in the commercial spectrum of applications in many sectors, with an approximate 30% share of the global enzyme market [7,8,9]. Amylases are widely used in the food, textile, detergent, and pharmaceutical industries [10,11,12]. α-Amylase can be obtained from several sources, such as plants, animals, fungi, and bacteria [3,13]. Bacterial α-amylases are prominent due to their low production costs, the ease of scale-up in fermentative production, and enzyme operational stability [14,15].

Thermostability is a desired feature for various industrial applications [16,17]. Thermophilic, mesophilic, and extremophilic bacteria are the primary sources of thermostable α-amylase enzymes [18,19]. Bacteria producing thermostable α-amylases are mainly derived from representatives of the *Bacillus* genus, including *B. subtilis*, *B. licheniformis*, and *B. amyloliquefaciens* [20,21,22]. α-Amylases are among the most versatile enzymes in industrial applications because of the abundant availability of starch as a substrate and their crucial role in producing cyclodextrins for the pharmaceutical industry [23]. The starch industry requires a thermostable α-amylase in the starch hydrolysis process, in which all three main steps of gelatinization, liquefaction, and saccharification are performed at a high temperature [24,25,26]. One of the most critical aspects of thermostable α-amylase enzymes in biotechnological processes is the use of elevated temperatures to reduce the risk of contamination by common microorganisms. In addition, high temperature increases the reaction rate and substrate diffusion to the active site [27,28]. These benefits highlight the importance of continued efforts to discover and isolate novel enzyme sources that can offer improved thermostability and other desirable traits for industrial applications [28,29].

Our study describes the cloning, expression, purification, and characterization of a thermostable α-amylase from *B. licheniformis* 104.K, isolated from Uzbekistan soils. With an optimal activity temperature of 90 °C, this enzyme represents a novel thermostable α-amylase producer in Uzbekistan. These findings provide valuable insights into thermostable enzymes and their potential for improving industrial processes involving starch-containing raw materials.

## 2. Materials and Methods

### 2.1. Isolation of Bacterial Strains

A soil sample from wheat-cultivated fields of the Kashkadarya region of Uzbekistan was used to isolate bacterial cultures that produced thermostable amylolytic enzymes. After isolation, the pure bacterial cultures were lyophilized and stored at 4–8 °C in a refrigerator for a long-term preservation over 12 years. The lyophilized bacterial cultures were dissolved in the nutrient broth and plated on nutrient agar. Individual colonies were transferred onto starch nutrient agar plates containing 1% starch, 2% agar, and 1.5% nutrient broth and incubated at 35 °C for 48 h. To determine the ability of the bacterial isolates to degrade starch, α-amylase activity was assessed by flooding the plates with a 10% iodine solution. Amylolytic activity was evaluated based on the formation of clear, colorless zones around the bacterial colonies against a dark blue background produced by the iodine–starch complex.

### 2.2. Identification of the Microorganism

Morphological–physiological and biochemical properties of the *Bacillus* spp. 104.K isolate were determined according to Bergey’s Manual [30]. Bacterial cells were stained with the Gram staining kit (Himedia, Mumbai, India) and observed under a light microscope. The *Bacillus* spp. 104.K isolate was cultured in nutrient broth at 35 °C for 24 h for genomic DNA isolation. Cells were harvested by centrifugation at 11,000 rpm and 4 °C for 5 min, and washed twice with a sterile 0.9% NaCl solution. Genomic DNA was extracted from the bacterial cultures using a modified Marmur method [31]. Bacterial cells were suspended in EDTA–saline, treated with lysozyme, followed by RNase A digestion and SDS lysis. After incubation at 65 °C, proteins were removed by repeated chloroform/isoamyl alcohol extraction. DNA was precipitated with cold isopropanol in the presence of sodium acetate, air-dried, and resuspended in low-TE buffer. The 16S rRNA region was amplified using the universal primers 27F (5′-AGAGTTTGATCMTGGCTCAG-3′), 517F (5′-GCCAGCAGCCGCGGTAA-3′), and 1492R (5′-TACGGTTACCTTGTTACGACTT-3′). Sequencing of the PCR products was performed using the Sanger sequencing method on the SeqStudio Genetic analyzer (Thermo Fisher Scientific, Waltham, MA, USA). Sequences were compared with GenBank data using the BLAST Local Alignment Search Tool 2.16.0+ [32]. In addition, this isolate was identified based on the proteomic profiles of ribosomal proteins, as mass spectrometry-based proteomics has revolutionized bacterial identification and is widely applied in both clinical and research settings [33,34]. Identification was performed using a Matrix-Assisted Laser Desorption/Ionization Time-of-Flight (MALDI-TOF) mass spectrometry system with the EXS2600 instrument (Zybio, Chongqing, China).

### 2.3. Cloning of the α-Amylase Gene

To isolate the α-amylase gene from genomic DNA of *B. licheniformis* 104.K, primers amyL_F (5′-CCAGGATCCATGAAACAACAAAAACGG-3′) and amyL_R (5′-CCACATATGGCTCTTCTATCTTTGAACAT-3′) were designed using SnapGene 7.2.1 (restriction sites underlined) based on the complete genome sequence of *B. licheniformis* strain SCDB 14 (GenBank: CP014842.1). The primers were synthesized and supplied by Integrated DNA Technologies (Leuven, Belgium). The α-amylase gene was amplified from the genomic DNA using Platinum Hot Start PCR 2X Master Mix (Invitrogen, Vilnius, Lithuania) on a C1000 Touch PCR thermal cycler (Bio-Rad, Hercules, CA, USA). The PCR amplification protocol consisted of three steps: (1) an initial denaturation at 95 °C for 4 min to ensure complete DNA strand separation; (2) 35 cycles of denaturation at 95 °C for 40 s, primer annealing at 55 °C for 30 s, and elongation at 72 °C for 90 s; and (3) a final elongation at 72 °C for 10 min. The PCR product and pUC18 vector (provided by Dr. A. Kachan) were prepared for cloning by digestion with the BamHI and NdeI restriction enzymes. Insertion of the *amyL* gene was screened by colony PCR and blue-white screening method [35]. Plasmids from two positive colonies were extracted using the Plasmid Extraction Kit (Qiagen, Hilden, Germany). The *amyL* gene was then subcloned into the pET-15b vector (Merck KGaA, Darmstadt, Germany) between the NdeI and BamHI restriction sites for high-level expression and simplified purification of the enzyme via affinity chromatography. For this, PCR primers amyL_F_Nde1 (5′-GCAACAACCATATGAAACAACAAAAACGGCTTTACGCC-3′) and amyL_R_BamH1 (5′-GATCGGATCCCTATCTTTGAACATAAATTGAAACCGACC-3′) were used (restriction sites underlined). Insert correctness was confirmed by Sanger sequencing using T7 promoter forward and T7 terminator reverse primers.

### 2.4. Expression and Purification of Recombinant α-Amylase

The pET-15b construct was transformed into *Escherichia coli* Rosetta (DE3) and *Escherichia coli* BL21 (DE3) (New England BioLabs, Ipswich, MA, USA) cells using the heat-shock transformation method. Transformants were cultured in LB medium supplemented with carbenicillin at 37 °C and 200 rpm. When the optical density of the culture reached 0.6 measured at 600 nm, isopropyl-β-thiogalactopyranoside (IPTG) was added to a final concentration of 0.2 mM. The cultures were then incubated at 30 °C for 4 h and subsequently at 16 °C overnight to optimize the expression conditions. Cells were harvested by centrifugation at 4700 rpm for 30 min at 4 °C using an Allegra X30R benchtop centrifuge equipped with swinging-bucket rotor (Beckmann Coulter, Brea, CA, USA), suspended in lysis buffer (300 mM NaCl, 50 mM Tris-HCl, pH 7.5, and lysozyme), ultrasonicated in an Ultrasonic Cell Disruptor UCD-1200 (Biobase Group, Jinan, China) five times with 30 s bursts, with 30 s intervals between each burst, followed by centrifugation of the obtained cell lysate at 11,000 rpm for 20 min at 4 °C using the fixed-angle rotor of the Allegra X30R benchtop centrifuge. The supernatant was transferred to a new tube, and the recombinant α-amylase was purified by immobilized metal affinity chromatography using a gravity nickel column system (Bio-Rad, Hercules, CA, USA). The nickel column was equilibrated with 10 mM imidazole in 300 mM NaCl and 50 mM Tris-HCl, pH 7.5. Recombinant α-amylase was eluted using 20–100 mM imidazole in a 300 mM NaCl and 50 mM Tris-HCl (pH 7.5) buffer solution. Eluted protein fractions were analyzed by 12% SDS-PAGE [36]. A homogeneous pure protein fraction with detected amylase activity was used in subsequent experiments.

### 2.5. Enzyme Assay

The α-amylase activity assay was performed using the 3,5-dinitrosalicylic acid (DNS) method with minor modifications [37]. α-Amylase activity was assessed at temperatures from 50 °C to 100 °C in a 0.6 ml reaction mixture that contained 0.54 mL of a 0.5% (*w*/*v*) solution of potato starch in 100 mM potassium phosphate buffer (pH 6.0), 5 mM CaCl_2_, and 0.06 mL of the enzyme solution. After an incubation for 10 min, the reaction was stopped by adding 1.8 mL of DNS reagent and boiling for 10 min in a water bath. The reaction mixture was cooled to room temperature, and the absorbance was measured at 540 nm. One unit of α-amylase activity was defined as the amount of enzyme that produces 1 µmol of reducing sugar per minute under the assay conditions. A glucose concentration range of 0–1 mg/mL was used to construct the standard curve.

### 2.6. Effects of Temperature on the Activity and Stability of Recombinant α-Amylase

The effect of temperature on the purified recombinant α-amylase activity was measured at various temperatures between 30 °C and 100 °C, with 10 °C intervals, in 100 mM phosphate buffer and pH 6.0. To determine the thermal stability, the enzyme was pre-incubated at different temperatures for 30 min in 100 mM Na-phosphate buffer at the optimum pH, and α-amylase activity was measured at 90 °C under standard enzyme assay conditions.

### 2.7. Effects of pH on the Activity and Stability of Recombinant α-Amylase

α-Amylase activity was determined in the pH range of 5–9 (with one-unit interval) at 90 °C, the experimentally defined optimum temperature for α-amylase activity. The pure enzyme was incubated for 1 h at 40 °C in different pH ranges in 100 mM Na-phosphate buffer to measure pH stability. Enzyme activity without prior incubation was set as 100%.

### 2.8. Effects of CaCl_2_ on the Activity and Thermal Stability of Recombinant α-Amylase

To determine the effects of Ca^2+^ ions on enzyme activity and thermostability, recombinant α-amylase was incubated at different temperatures in a 5 mM CaCl_2_ solution in Na-phosphate buffer (100 mM, pH 6.0). The enzyme was incubated at different temperatures for 30 min, followed by enzyme activity measurement under the optimum conditions. Enzyme activity without prior incubation was set as 100%.

### 2.9. Bioinformatics and Structural Analyses

The 3D structure of *B. licheniformis* 104.K α-amylase was predicted using AlphaFold3 [38]. Five models were generated using one copy of the protein sequence as the input. We selected the best prediction based on the predicted local distance difference test (plDDT) score. Amylase was classified using the dbCAN3 CAZy metaserver (https://bcb.unl.edu/dbCAN2_obsolete/, accessed on 1 January 2025) [4]. Enzyme domains A, B, and C were identified using the NCBI Conserved Domain Database [39] and the InterPro Database 105.0 [40]. The *amyL* gene was translated using the ExPASy Translate tool (https://web.expasy.org/translate/, accessed on 1 January 2025), and the theoretical molecular mass was predicted with the ProtParam tool (https://web.expasy.org/protparam/, accessed on 1 January 2025) [41]. Multiple sequence alignment was performed using the Clustal Omega program (https://www.ebi.ac.uk/jdispatcher/msa/clustalo?stype=protein, accessed on 1 January 2025) [42]. SnapGene software (www.snapgene.com, accessed on 1 May 2024) was used for primer design and cloning simulation. A phylogenetic tree was generated using MEGA 11 software [43]. The 3D structure of amylase was visualized using PyMOL 3.0.

## 3. Results

### 3.1. Bacterial Strain Identification and Cloning of the α-Amylase Gene

**Figure 2:**
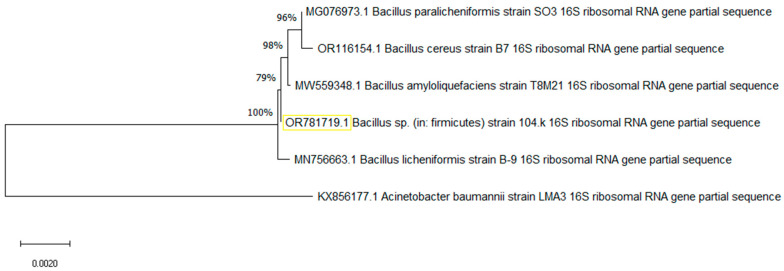
The phylogenetic tree of *B. licheniformis* 104.K (GenBank: OR781719.1) and related strains was constructed using MEGA 11 software. The yellow-framed accession OR781719.1 is the query sequence, phylogenetically grouped within *Bacillus* species, closely related to *B. licheniformis*. Multiple sequence alignment was performed using the MUSCLE algorithm, and the phylogenetic tree was generated using the neighbor-joining method. Bootstrap analysis with 1000 replicates was conducted to evaluate the reliability of the tree topology.

First, the *Bacillus* spp. 104.K isolate was inoculated on a 1% starch-containing agar plate to test α-amylase activity, as shown in Supporting Information, Figure S1. Clearance zones of the blue color with a 7–8 mm diameter around the colonies were detected after incubation at 35 °C for 48 h, which showed starch hydrolysis and confirmed the α-amylase activity of the *Bacillus* spp. 104.K strain. Based on the morphological–physiological and biochemical properties, and the sequence of the conserved 16S rRNA gene region (99.58% sequence identity), and MALDI-TOF MS data (2.21 points with a green color), the *Bacillus* spp. 104.K isolate was identified as *Bacillus licheniformis* and its phylogenetic tree was created based on 16S rRNA gene homology using neighbor-joining method in MEGA 11 software (Figure 2). The *amyL* gene encoding α-amylase was successfully amplified from the genomic DNA of the *B. licheniformis* 104.K strain.

### 3.2. Expression and Purification of Recombinant α-Amylase

**Figure 3:**
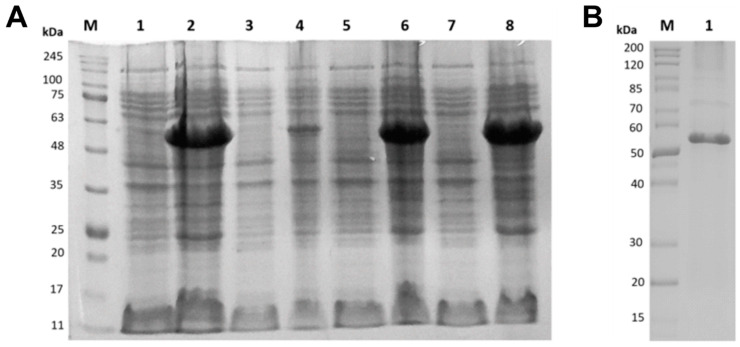
(**A**) Image of the 12% SDS-PAGE gel containing the recombinant α-amylase of *B. licheniformis* 104.K purified by affinity chromatography. Lane M: protein marker (Sigma-Aldrich, BLUeye Prestained Protein Ladder). Lanes 1 and 2: *E. coli* BL21 before and after induction at 16 °C with overnight incubation. Lanes 3 and 4: *E. coli* BL21 before and after induction at 30 °C with 4 h of incubation. Lanes 5 and 6: *E. coli* Rosetta before and after induction at 16 °C with overnight incubation. Lanes 7 and 8: *E. coli* Rosetta before and after induction at 30 °C with 4 h of incubation. (**B**) Lane M: protein marker (PageRuler Unstained Protein Ladder). Lane 1: Ni-NTA affinity-purified recombinant α-amylase expressed in *E. coli* Rosetta cells.

The *amyL* gene was subcloned into the pET-15b expression vector harboring an N-terminal His-tag (6x-His) before the cloning site. The recombinant His_6_-a-amylase was expressed in *E. coli* BL21 (DE3) and Rosetta (DE3) and appeared in the induced cell lysate at ~60 kDa on a 12% SDS-PAGE gel (Figure 3A), in accordance with the theoretical molecular mass (58.492kDa) predicted by the ProtParam server. His_6_-a-amylase was purified from Rosetta cells using Ni-NTA chromatography (Figure 3B). The concentration of purified α-amylase was 0.1 mg/mL.

### 3.3. Effects of Temperature, pH, and Ca^2+^ Ions on α-Amylase Activity and Stability

**Figure 4:**
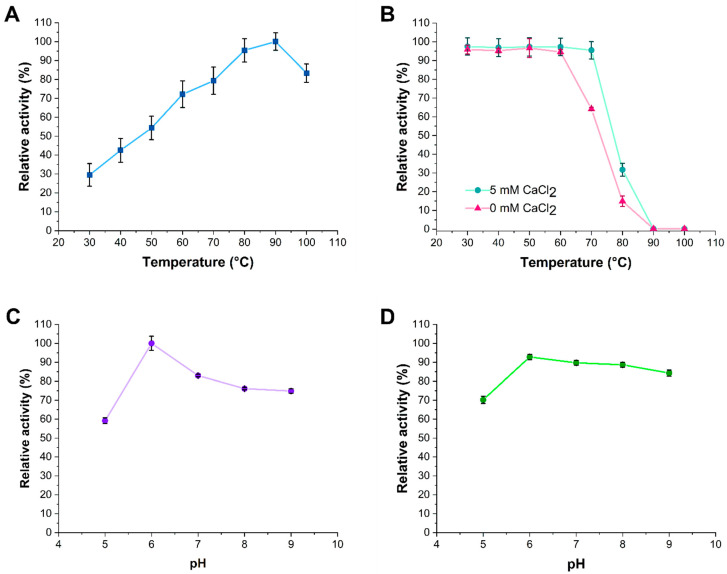
*B. licheniformis* 104.K α-amylase enzyme activity and stability are affected by different factors. (**A**) Effects of temperature on α-amylase activity. (**B**) α-Amylase thermostability in the presence or absence of 5 mM calcium chloride. (**C**) Effects of pH on α-amylase activity at 90 °C. (**D**) Effects of pH on α-amylase stability. The pH stability was determined at 90 °C in Na-phosphate buffer (100 mM) by varying the pH values. The enzyme was preincubated in the buffer at 40 °C for 1 h, and residual activity was determined. The activity of the enzyme before incubation was taken as 100%. The average and standard deviation of the measured data of three parallel measurements are shown, with trend lines connecting the data points.

The specific activity of recombinant α-amylase under the optimum assay conditions (pH 6.0 and 90 °C) was 1630 ± 0.4 U/mg (163 ± 0.4 U/mL or 2.80 × 10^−6^ kat/mL). The temperature dependence analysis of α-amylase activity demonstrated its capacity to function at high temperatures. α-Amylase activity increased at temperatures ranging from 30 °C to 90 °C and started to decrease slowly at 100 °C. The temperature of maximal activity was around 90 °C, while at 80 °C and 100 °C, the recombinant α-amylase showed 95% and 83% relative activity, respectively (Figure 4A). The thermal stability profile of the α-amylase showed that the enzyme was stable from 30 °C to 60 °C. The enzyme retained about 100% of its initial activity after 30 min of incubation from 30 °C to 60 °C. However, the enzyme retained 63% of its activity after 30 min of incubation at 70 °C and 11% after incubation at 80 °C, whereas 30 min of incubation at 90 °C completely inactivated the enzyme (Figure 4B). Moreover, adding 5 mM CaCl_2_ to the reaction mixture enhanced the recombinant α-amylase’s thermal stability. In the presence of CaCl_2_ at 70 °C, the enzyme lost only 5% of its initial activity after 30 min of incubation. The addition of 5 mM CaCl_2_ slightly increased the enzyme activity (Figure 4B).

The effect of pH was determined in the pH range from 5.0 to 9.0, and pH 6.0 was found as optimum for purified recombinant α-amylase. Increasing or decreasing the pH value slightly reduced the activity of the enzyme. The enzyme displayed the lowest activity (59%) within the assessed pH range at pH 5.0. In the pH range from 7.0 to 9.0, the activity decreased from 83% to 74% (Figure 4C). To assess the effect of pH on enzyme stability, the enzyme was preincubated in buffer solutions at 40 °C for 1 h. After this preincubation, the enzyme retained 69% of its initial activity measured at 90 °C at pH 5.0, while at pH ranges from 6.0 to 9.0, the enzyme activity gradually decreased from 92% to 85% (Figure 4D).

### 3.4. Phylogenetic and Computational Analyses

**Figure 5:**
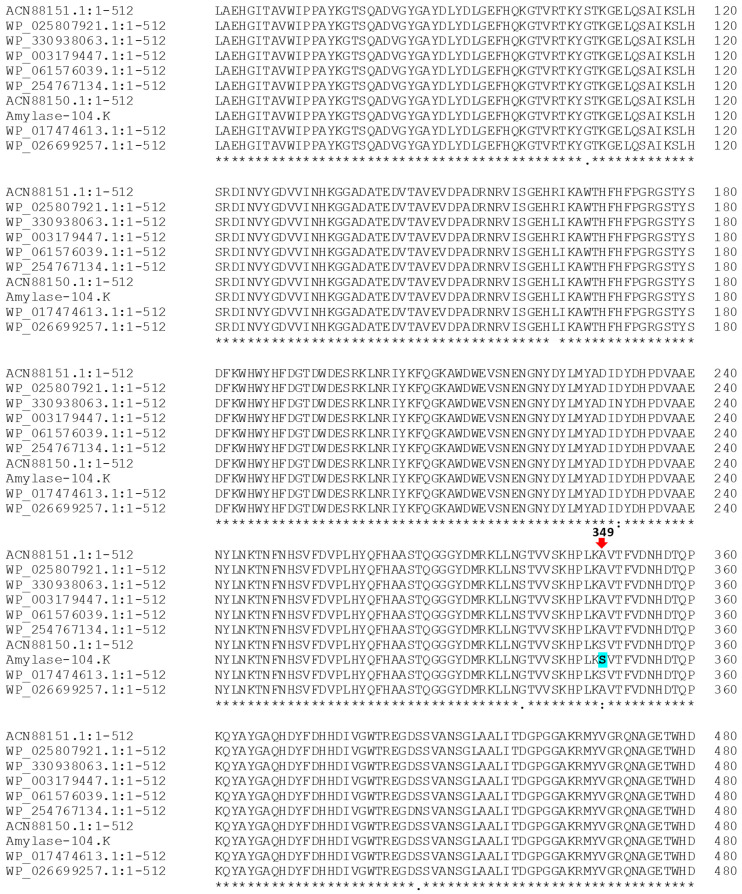
Sequence alignment of the thermostable α-amylase (Amylase-104.K) from *B. licheniformis* 104.K with sequences from the NCBI protein database. A key mutation at position 349 is highlighted in blue, showcasing the amino acid variation unique to the amylase from the 104.K strain (“*”—identical residues; “:”—strongly similar residues; “.”—weakly similar residues). The alignment was generated using Clustal Omega software.

The amino acid sequence corresponding to the *amyL* gene of the identified *B. licheniformis* 104.K was compared with NCBI protein BLAST homologs (Figure 5, the complete sequence alignment is provided in the Supporting Information, Figure S2). Most of these homologs correspond to unpublished and uncharacterized amylases except for the one described by Liu et al. [44]. α-Amylase was classified using the automated Carbohydrate-Active Enzyme Annotation (dbCAN3) web server [5]. The results indicated that the enzyme belongs to the GH13 family, specifically the GH13_5 subfamily, which is known for its broad substrate specificity, particularly for starch hydrolysis by cleaving alpha-1,4-glycosidic bonds in starch and glycogen [45,46]. This process is facilitated by specialized binding sites outside the enzyme’s active site [6].

**Figure 6:**
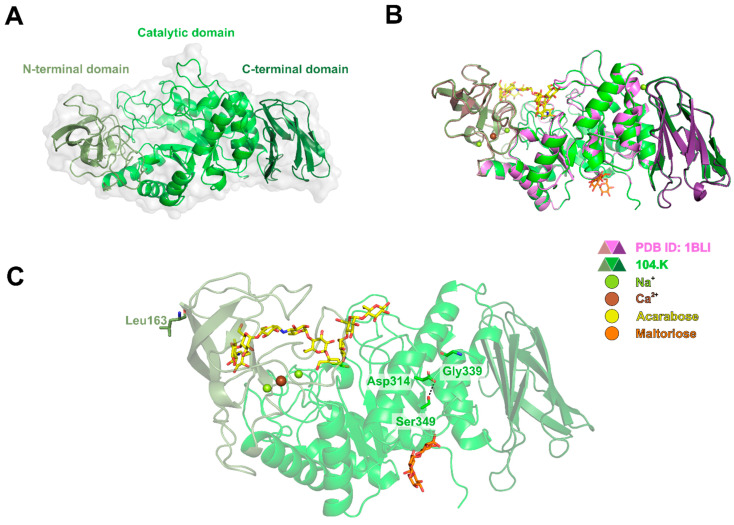
AlphaFold structure of *B. licheniformis* 104.K α-amylase. (**A**) Overall structure of processed *B. licheniformis* 104.K α-amylase. The structure is shown as a cartoon and a partially transparent surface; the three main domains are highlighted in three shades of green. (**B**) Structural comparison with active α-amylase (PDB ID: 1BLI), shown as cartoon colored in various shades of purple. Catalytic metal cofactors are shown as lime and brown spheres for Na^+^ and Ca^2+^, respectively. The enzyme active site is designated with superimposed acarbose (PDB ID: 1E3Z) shown as sticks with atomic coloring (C, yellow; O, red; N, blue) and an auxiliary starch binding site by a superimposed maltotriose (PDB ID: 1E40) also shown as sticks with atomic coloring (C, orange; O, red; N, blue). (**C**) Predicted structure *B. licheniformis* 104.K α-amylase highlighting the residue differences compared to *B. licheniformis* α-amylase (UniProt ID: P06278). The different residues are shown as sticks, and potential interactions contributing to better thermostability are indicated as black dashed lines.

Next, we performed a sequence and structure-based comparison of 104.K α-amylase with the *B. licheniformis* α-amylase entries. Sequence comparison of 104.K α-amylase with the standard α-amylase isolate (UniProt: P06278) (Supporting Information, Figure S3) revealed three residue substitutions dispersed in the protein sequence. To assess their structural effects, we created an AlphaFold3 model, displaying the well-described α-amylase fold composed of an N-terminal domain, a (β/α)_8_ barrel folded catalytic domain, and a C-terminal Greek key motif domain [39] (Figure 6A). We superimposed this with a *B. licheniformis* α-amylase in an active conformation (PDB ID: 1BLI) to highlight substrate binding sites (Figure 6B). Based on the structural analysis, two residue substitutions (Arg169Leu and Ser339Gly) are surface localized in the N-terminal and catalytic domains, respectively, which may modulate enzyme thermal stability through their surface polarity effect. An additional Ala349Ser residue substitution introduces a polar contact with Asp-314, which could provide a subtle conformational effect in the vicinity of the auxiliary starch binding site indicated by a superimposed maltotriose (PDB ID: 1E40), potentially modulating bulk starch substrate binding (Figure 6C) [47].

## 4. Discussion

This study investigated the optimal temperature, pH, and calcium ion requirements for the recombinant α-amylase derived from *B. licheniformis* 104.K. Previous studies have shown that α-amylases from *Bacillus* species typically exhibit temperature optima between 70 °C and 80 °C and show maximal activity at pH values ranging from 6.0 to 7.0 [46]. Based on this, we evaluated the enzyme’s activity across a temperature range of 30 °C to 100 °C and pH range of 4.0 to 9.0. Notably, the temperature optimum of the recombinant α-amylase was higher than that reported for α-amylases in previous studies [8,48,49,50]. Calcium ion is an essential metal cofactor that enhances the structural stability of most α-amylases [28,51]. Consistently, our findings indicate that the recombinant α-amylase from *B. licheniformis* 104.K requires calcium ions for its thermal stability. Structural predictions based on the computational analysis (Figure 6), together with experimental data, confirmed this requirement. The addition of 5 mM CaCl_2_ significantly enhanced the enzyme’s thermal stability (Figure 4B).

The thermostable α-amylase from *B. licheniformis* 104.K demonstrates distinct characteristics compared to the well-characterized thermostable and acid-resistant alpha-amylase precursor (GenBank: ACN88151.1) [44]. The acid-resistant alpha-amylase precursor exhibits optimal activity at 95 °C and retains significant stability, with minimal activity loss after 2 h of incubation at 80 °C. In contrast, the thermostable α-amylase from *B. licheniformis* 104 K has an optimal activity at 90 °C but loses 89% of its activity within 30 min at 80 °C. This indicates a critical need to enhance the enzyme’s thermostability for broader industrial utility. These differences in stability are influenced by amino acid variations, as evidenced by studies showing that mutagenesis of specific residues (107Gly → Ser, 163Leu → Arg, and 349Ser → Ala; see the Supporting Information, Figure S4) can significantly improve thermostability and acid resistance. This aligns with the necessity of employing enzyme engineering techniques, including site-directed mutagenesis and rational design, to tailor α-amylase properties for industrial demands.

Our findings highlight the isolation of *B. licheniformis* 104.K from underexplored soils in Uzbekistan, demonstrating its potential as a valuable source of thermostable α-amylase for industrial application. The enzyme exhibits high activity at elevated temperatures, making it well-suited for applications such as starch liquefaction in the food industry and detergent formulation [24]. Additionally, it shows promise in second-generation biofuel production, where enzymatic hydrolysis of non-edible agricultural wastes rich in cellulose, hemicellulose, and starch is critical. Thermostable α-amylases play a key role in enhancing starch degradation during saccharification, thereby boosting bioethanol yields [52]. Although the thermostability of this enzyme is somewhat lower than that of extensively engineered α-amylases [53,54], it remains a promising candidate for further optimization through protein engineering. The unique origin of *B. licheniformis* 104.K underscores the value of exploring untapped microbial diversity to discover novel enzymes with industrial potential.

## 5. Conclusions

A bacterial strain with amylolytic properties was isolated from under-represented soils of Uzbekistan and identified as *B. licheniformis* 104.K based on morphological, molecular, genetic, and proteomic compositions.

The α-amylase gene from this strain was successfully cloned into cloning and expression plasmids and expressed in *E. coli*. The recombinant enzyme was purified and characterized, exhibiting excellent thermal stability and catalytic activity.

Phylogenetic analysis revealed that the enzyme belongs to the GH13_5 subfamily of α-amylases and features unique amino acid variations in its catalytic domain, influencing its distinct enzymatic properties.

The results indicate that the α-amylase derived from *B. licheniformis* 104.K has great potential for enhancing thermostability and catalytic activity in protein engineering applications. It is suitable for industrial applications such as processing starch-containing raw materials.

## Data Availability

The datasets generated and/or analyzed during the current study are available in the GENBANK repository, accession numbers OR781719.1 and PQ285381.1.

## Supplementary Materials

The following supporting information can be downloaded at https://www.mdpi.com/article/10.3390/microorganisms13081757/s1: Figure S1. *B. licheniformis* 104.K α-amylase enzyme activity assay on agar plates. Hydrolytic zone of native *B. licheniformis* 104.K on a 1% starch-containing agar plate. The plate was stained with 10% iodine solution. Figure S2. Sequence alignment of the thermostable α-amylase (Amylase-104.K) from *B. licheniformis* 104.K with sequences from the NCBI protein database. A key mutation at position 349 is highlighted in blue, showcasing the amino acid variation unique to the amylase from the 104.K strain. The alignment was generated using Clustal Omega software. Figure S3. Sequence alignment of the thermostable α-amylase (Amylase-104.K) from *B. licheniformis* 104.K and the UniProt-annotated *B. licheniformis* α-amylase sequence (UniProt ID: P06278). Amino acid variation positions are shown in bold and highlighted in green. The alignment was generated using Clustal Omega. Figure S4. Sequence alignment of the thermostable α-amylase (Amylase-104.K) from *B. licheniformis* 104.K and a synthetic α-amylase construct (GenBank ID: ACN88151.1). Amino acid variation positions are shown in bold and highlighted in cyan. The alignment was generated using Clustal Omega.
