# Supplementary material for: Characterization of a Thermostable α-Amylase from Bacillus licheniformis 104.K for Industrial Applications
Source: Microorganisms. 2025 Jul 28;13(8):1757. doi: 10.3390/microorganisms13081757 (PMC12388735; doi:10.3390/microorganisms13081757)
Supplement: Supplementary file 1 [file microorganisms-13-01757-s001.zip › microorganisms-3683030-supplementary.pdf]

SUPPORTING INFORMATION

Characterization of a Thermostable α-Amylase from *Bacillus licheniformis* 104. K for Industrial Applications

Askar Kholikov[1, 2], Khushnut Vokhidov[1], Azizjon Murtozoyev[1], Zoé S. Tóth[3,4], Gergely N. Nagy[2, 3], Beáta G. Vértessy[2, 3, *], Akhmadzhan Makhsumkhanov[1,*]

[1]Institute of Microbiology, Uzbekistan Academy of Sciences, Tashkent, Uzbekistan

[2]Department of Applied Biotechnology and Food Science, Faculty of Chemical Technology and Biotechnology, Budapest University of Technology and Economics, Budapest, Hungary

[3]Institute of Molecular Life Sciences, HUN-REN Research Centre for Natural Sciences, Budapest, Hungary

[4]Doctoral School of Biology, Institute of Biology, ELTE Eötvös Loránd University, Budapest, Hungary

Correspondence: vertessy.beata@ttk.hu (B.G.V.); amakhsumkhanov@gmail.com (A.M.)

Supporting Figures

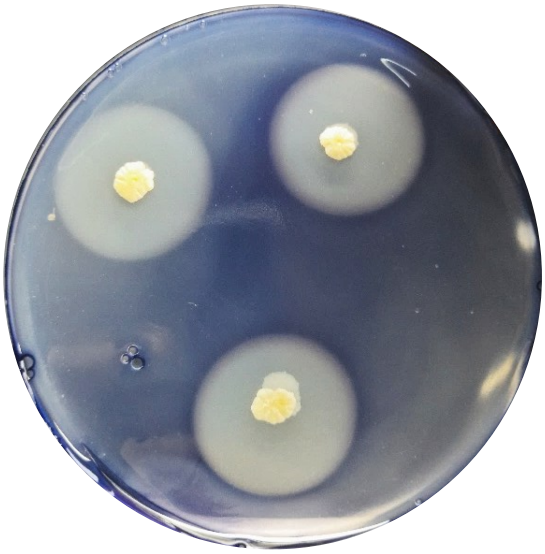

Figure S1. ***B. licheniformis*** **104.K α-amylase enzyme activity assay on agar plates.** Hydrolytic zone of native *B. licheniformis* 104.K on a 1% starch-containing agar plate. The plate was stained with 10% iodine solution.

```
ACN88151.1:1-512          MKQQKRLYARLLTLLFALIFLLPHSAAAAANLNGTLMQYFEWYMPNDGQHWKRLQNDSAY   60
WP_025807921.1:1-512      MKQQKRLYARLLTLLFALIFLLPHSAAAAANLNGTLMQYFEWYMPNDGQHWKRLQNDSAY   60
WP_330938063.1:1-512      MKQQKRLYARLLTLLFALIFLLPHSAAAAANLNGTLMQYFEWYMPNDGQHWKRLQNDSAY   60
WP_003179447.1:1-512      MKQQKRLYARLLTLLFALIFLLPHSAAAAANLNGTLMQYFEWYMPNDGQHWKRLQNDSAY   60
WP_061576039.1:1-512      MKQQKRLYARLLTLLFALIFLLPHSAAAAANLNGTLMQYFEWYMPNDGQHWKRLQNDSAY   60
WP_254767134.1:1-512      MKQQKRLYARLLTLLFALIFLLPHSAAAAANLNGTLMQYFEWYMPNDGQHWKRLQNDSAY   60
ACN88150.1:1-512          MKQQKRLYARLLTLLFALIFLLPHSAAAAANLNGTLMQYFEWYMPNDGQHWKRLQNDSAY   60
Amylase-104.K             MKQQKRLYARLLTLLFALIFLLPHSAAAAANLNGTLMQYFEWYMPNDGQHWKRLQNDSAY   60
WP_017474613.1:1-512      MKQQKRLYARLLTLLFALIFLLPHSAAAAANLNGTLMQYFEWYMPNDGQHWKRLQNDSAY   60
WP_026699257.1:1-512      MKQQKRLYARLLTLLFALIFLLPHSAAAAANLNGTLMQYFEWYMPNDGQHWKRLQNDSAY   60
                          ************************************************************

ACN88151.1:1-512          LAEHGITAVWIPPAYKGTSQADVGYGAYDLYDLGEFHQKGTVRTKYSTKGELQSAIKSLH   120
WP_025807921.1:1-512      LAEHGITAVWIPPAYKGTSQADVGYGAYDLYDLGEFHQKGTVRTKYGTKGELQSAIKSLH   120
WP_330938063.1:1-512      LAEHGITAVWIPPAYKGTSQADVGYGAYDLYDLGEFHQKGTVRTKYGTKGELQSAIKSLH   120
WP_003179447.1:1-512      LAEHGITAVWIPPAYKGTSQADVGYGAYDLYDLGEFHQKGTVRTKYGTKGELQSAIKSLH   120
WP_061576039.1:1-512      LAEHGITAVWIPPAYKGTSQADVGYGAYDLYDLGEFHQKGTVRTKYGTKGELQSAIKSLH   120
WP_254767134.1:1-512      LAEHGITAVWIPPAYKGTSQADVGYGAYDLYDLGEFHQKGTVRTKYGTKGELQSAIKSLH   120
ACN88150.1:1-512          LAEHGITAVWIPPAYKGTSQADVGYGAYDLYDLGEFHQKGTVRTKYSTKGELQSAIKSLH   120
Amylase-104.K             LAEHGITAVWIPPAYKGTSQADVGYGAYDLYDLGEFHQKGTVRTKYGTKGELQSAIKSLH   120
WP_017474613.1:1-512      LAEHGITAVWIPPAYKGTSQADVGYGAYDLYDLGEFHQKGTVRTKYGTKGELQSAIKSLH   120
WP_026699257.1:1-512      LAEHGITAVWIPPAYKGTSQADVGYGAYDLYDLGEFHQKGTVRTKYGTKGELQSAIKSLH   120
                          **********************************************.*************

ACN88151.1:1-512          SRDINVYGDVVINHKGGADATEDVTAVEVDPADRNRVISGEHRIKAWTHFHFPGRGSTYS   180
WP_025807921.1:1-512      SRDINVYGDVVINHKGGADATEDVTAVEVDPADRNRVISGEHRIKAWTHFHFPGRGSTYS   180
WP_330938063.1:1-512      SRDINVYGDVVINHKGGADATEDVTAVEVDPADRNRVISGEHLIKAWTHFHFPGRGSTYS   180
WP_003179447.1:1-512      SRDINVYGDVVINHKGGADATEDVTAVEVDPADRNRVISGEHRIKAWTHFHFPGRGSTYS   180
WP_061576039.1:1-512      SRDINVYGDVVINHKGGADATEDVTAVEVDPADRNRVISGEHLIKAWTHFHFPGRGSTYS   180
WP_254767134.1:1-512      SRDINVYGDVVINHKGGADATEDVTAVEVDPADRNRVISGEHLIKAWTHFHFPGRGSTYS   180
ACN88150.1:1-512          SRDINVYGDVVINHKGGADATEDVTAVEVDPADRNRVISGEHLIKAWTHFHFPGRGSTYS   180
Amylase-104.K             SRDINVYGDVVINHKGGADATEDVTAVEVDPADRNRVISGEHLIKAWTHFHFPGRGSTYS   180
WP_017474613.1:1-512      SRDINVYGDVVINHKGGADATEDVTAVEVDPADRNRVISGEHLIKAWTHFHFPGRGSTYS   180
WP_026699257.1:1-512      SRDINVYGDVVINHKGGADATEDVTAVEVDPADRNRVISGEHLIKAWTHFHFPGRGSTYS   180
                          ****************************************** *****************

ACN88151.1:1-512          DFKWHWYHFDGTDWDESRKLNRIYKFQGKAWDWEVSNENGNYDYLMYADIDYDHPDVAAE   240
WP_025807921.1:1-512      DFKWHWYHFDGTDWDESRKLNRIYKFQGKAWDWEVSNENGNYDYLMYADIDYDHPDVAAE   240
WP_330938063.1:1-512      DFKWHWYHFDGTDWDESRKLNRIYKFQGKAWDWEVSNENGNYDYLMYADINYDHPDVAAE   240
WP_003179447.1:1-512      DFKWHWYHFDGTDWDESRKLNRIYKFQGKAWDWEVSNENGNYDYLMYADIDYDHPDVAAE   240
WP_061576039.1:1-512      DFKWHWYHFDGTDWDESRKLNRIYKFQGKAWDWEVSNENGNYDYLMYADIDYDHPDVAAE   240
WP_254767134.1:1-512      DFKWHWYHFDGTDWDESRKLNRIYKFQGKAWDWEVSNENGNYDYLMYADIDYDHPDVAAE   240
ACN88150.1:1-512          DFKWHWYHFDGTDWDESRKLNRIYKFQGKAWDWEVSNENGNYDYLMYADIDYDHPDVAAE   240
Amylase-104.K             DFKWHWYHFDGTDWDESRKLNRIYKFQGKAWDWEVSNENGNYDYLMYADIDYDHPDVAAE   240
WP_017474613.1:1-512      DFKWHWYHFDGTDWDESRKLNRIYKFQGKAWDWEVSNENGNYDYLMYADIDYDHPDVAAE   240
WP_026699257.1:1-512      DFKWHWYHFDGTDWDESRKLNRIYKFQGKAWDWEVSNENGNYDYLMYADIDYDHPDVAAE   240
                          **************************************************:*********

ACN88151.1:1-512          IKRWGTWYANELQLDGFRLDAVKHIKFSFLRDWVNHVREKTGKEMFTVAEYWQNDLGALE   300
WP_025807921.1:1-512      IKRWGTWYANELQLDGFRLDAVKHIKFSFLRDWVNHVREKTGKEMFTVAEYWQNDLGALE   300
WP_330938063.1:1-512      IKRWGTWYANELQLDGFRLDAVKHIKFSFLRDWVNHVREKTGKEMFTVAEYWQNDLGALE   300
WP_003179447.1:1-512      IKRWGTWYANELQLDGFRLDAVKHIKFSFLRDWVNHVREKTGKEMFTVAEYWQNDLGALE   300
WP_061576039.1:1-512      IKRWGTWYANELQLDGFRLDAVKHIKFSFLRDWVNHVREKTGKEMFTVAEYWQNDLGALE   300
WP_254767134.1:1-512      IKRWGTWYANELQLDGFRLDAVKHIKFSFLRDWVNHVREKTGKEMFTVAEYWQNDLGALE   300
ACN88150.1:1-512          IKRWGTWYANELQLDGFRLDAVKHIKFSFLRDWVNHVREKTGKEMFTVAEYWQNDLGALE   300
Amylase-104.K             IKRWGTWYANELQLDGFRLDAVKHIKFSFLRDWVNHVREKTGKEMFTVAEYWQNDLGALE   300
WP_017474613.1:1-512      IKRWGTWYANELQLDGFRLDAVKHIKFSFLRDWVNHVREKTGKEMFTVAEYWQNDLGALE   300
WP_026699257.1:1-512      IKRWGTWYANELQLDGFRLDAVKHIKFSFLRDWVNHVREKTGKEMFTVAEYWQNDLGALE   300
                          ************************************************************

ACN88151.1:1-512          NYLNKTNFNHSVFDVPLHYQFHAASTQGGGYDMRKLLNGTVVSKHPLKAVTFVDNHDTQP   360
WP_025807921.1:1-512      NYLNKTNFNHSVFDVPLHYQFHAASTQGGGYDMRKLLNSTVVSKHPLKAVTFVDNHDTQP   360
WP_330938063.1:1-512      NYLNKTNFNHSVFDVPLHYQFHAASTQGGGYDMRKLLNSTVVSKHPLKAVTFVDNHDTQP   360
WP_003179447.1:1-512      NYLNKTNFNHSVFDVPLHYQFHAASTQGGGYDMRKLLNGTVVSKHPLKAVTFVDNHDTQP   360
WP_061576039.1:1-512      NYLNKTNFNHSVFDVPLHYQFHAASTQGGGYDMRKLLNSTVVSKHPLKAVTFVDNHDTQP   360
WP_254767134.1:1-512      NYLNKTNFNHSVFDVPLHYQFHAASTQGGGYDMRKLLNGTVVSKHPLKAVTFVDNHDTQP   360
```

```
ACN88150.1:1-512        NYLNKTNFNHSVFDVPLHYQFHAASTQGGGYDMRKLLNGTVVSKHPLKSVTFVDNHDTQP   360
Amylase-104.K           NYLNKTNFNHSVFDVPLHYQFHAASTQGGGYDMRKLLNGTVVSKHPLKSVTFVDNHDTQP   360
WP_017474613.1:1-512    NYLNKTNFNHSVFDVPLHYQFHAASTQGGGYDMRKLLNGTVVSKHPLKSVTFVDNHDTQP   360
WP_026699257.1:1-512    NYLNKTNFNHSVFDVPLHYQFHAASTQGGGYDMRKLLNGTVVSKHPLKAVTFVDNHDTQP   360
                        **************************************.*********:***********

ACN88151.1:1-512        GQSLESTVQTWFKPLAYAFILTRESGYPQVFYGDMYGTKGDSQREIPALKHKIEPILKAR   420
WP_025807921.1:1-512    GQSLESTVQTWFKPLAYAFILTRESGYPQVFYGDMYGTKGDSQREIPALKHKIEPILKAR   420
WP_330938063.1:1-512    GQSLESTVQTWFKPLAYAFILTRESGYPQVFYGDMYGTKGDSQREIPALKHKIEPILKAR   420
WP_003179447.1:1-512    GQSLESTVQTWFKPLAYAFILTRESGYPQVFYGDMYGTKGDSQREIPALKHKIEPILKAR   420
WP_061576039.1:1-512    GQSLESTVQTWFKPLAYAFILTRESGYPQVFYGDMYGTKGDSQREIPALKHKIEPILKAR   420
WP_254767134.1:1-512    GQSLESTVQTWFKPLAYAFILTRESGYPQVFYGDMYGTKGDSQREIPALKHKIEPILKAR   420
ACN88150.1:1-512        GQSLESTVQTWFKPLAYAFILTRESGYPQVFYGDMYGTKGDSQREIPALKHKIEPILKAR   420
Amylase-104.K           GQSLESTVQTWFKPLAYAFILTRESGYPQVFYGDMYGTKGDSQREIPALKHKIEPILKAR   420
WP_017474613.1:1-512    GQSLESTVQTWFKPLAYAFILTRESGYPQVFYGDMYGTKGDSQREIPALKHKIEPILKAR   420
WP_026699257.1:1-512    GQSLESTVQTWFKPLAYAFILTRESGYPQVFYGDMYGTKGDSQREIPALKHKIEPILKAR   420
                        ************************************************************

ACN88151.1:1-512        KQYAYGAQHDYFDHHDIVGWTREGDSSVANSGLAALITDGPGGAKRMYVGRQNAGETWHD   480
WP_025807921.1:1-512    KQYAYGAQHDYFDHHDIVGWTREGDSSVANSGLAALITDGPGGAKRMYVGRQNAGETWHD   480
WP_330938063.1:1-512    KQYAYGAQHDYFDHHDIVGWTREGDSSVANSGLAALITDGPGGAKRMYVGRQNAGETWHD   480
WP_003179447.1:1-512    KQYAYGAQHDYFDHHDIVGWTREGDSSVANSGLAALITDGPGGAKRMYVGRQNAGETWHD   480
WP_061576039.1:1-512    KQYAYGAQHDYFDHHDIVGWTREGDSSVANSGLAALITDGPGGAKRMYVGRQNAGETWHD   480
WP_254767134.1:1-512    KQYAYGAQHDYFDHHDIVGWTREGDNSVANSGLAALITDGPGGAKRMYVGRQNAGETWHD   480
ACN88150.1:1-512        KQYAYGAQHDYFDHHDIVGWTREGDSSVANSGLAALITDGPGGAKRMYVGRQNAGETWHD   480
Amylase-104.K           KQYAYGAQHDYFDHHDIVGWTREGDSSVANSGLAALITDGPGGAKRMYVGRQNAGETWHD   480
WP_017474613.1:1-512    KQYAYGAQHDYFDHHDIVGWTREGDSSVANSGLAALITDGPGGAKRMYVGRQNAGETWHD   480
WP_026699257.1:1-512    KQYAYGAQHDYFDHHDIVGWTREGDSSVANSGLAALITDGPGGAKRMYVGRQNAGETWHD   480
                        *************************.**********************************

ACN88151.1:1-512        ITGNRSEPVVINSEGWGEFHVNGGSVSIYVQR 512
WP_025807921.1:1-512    ITGNRSEPVVINSEGWGEFHVNGGSVSIYVQR 512
WP_330938063.1:1-512    ITGNRSEPVVINSEGWGEFHVNGGSVSIYVQR 512
WP_003179447.1:1-512    ITGNRSEPVVINSEGWGEFHVNGGSVSIYVQR 512
WP_061576039.1:1-512    ITGNRSEPVVINSEGWGEFHVNGGSVSIYVQR 512
WP_254767134.1:1-512    ITGNRSEPVVINSEGWGEFHVNGGSVSIYVQR 512
ACN88150.1:1-512        ITGNRSEPVVINSEGWGEFHVNGGSVSIYVQR 512
Amylase-104.K           ITGNRSEPVVINSEGWGEFHVNGGSVSIYVQR 512
WP_017474613.1:1-512    ITGNRSEPVVINSEGWGEFHVNGGSVSIYVQR 512
WP_026699257.1:1-512    ITGNRSEPVVINSEGWGEFHVNGGSVSIYVQR 512
                        ********************************
```

Figure S2. **Sequence alignment of the thermostable α-amylase (Amylase-104.K) from *B. licheniformis* 104.K with sequences from the NCBI protein database.** A key mutation at position 349 is highlighted in blue, showcasing the amino acid variation unique to the amylase from the 104.K strain. The alignment was generated using Clustal Omega software.

```
P06278      MKQQKRLYARLLTLLFALIFLLPHSAAAAANLNGTLMQYFEWYMPNDGQHWKRLQNDSAY  60
104.K       MKQQKRLYARLLTLLFALIFLLPHSAAAAANLNGTLMQYFEWYMPNDGQHWKRLQNDSAY  60
            ************************************************************

P06278      LAEHGITAVWIPPAYKGTSQADVGYGAYDLYDLGEFHQKGTVRTKYGTKGELQSAIKSLH  120
104.K       LAEHGITAVWIPPAYKGTSQADVGYGAYDLYDLGEFHQKGTVRTKYGTKGELQSAIKSLH  120
            ************************************************************

P06278      SRDINVYGDVVINHKGGADATEDVTAVEVDPADRNRVISGEHRIKAWTHFHFPGRGSTYS  180
104.K       SRDINVYGDVVINHKGGADATEDVTAVEVDPADRNRVISGEHLIKAWTHFHFPGRGSTYS  180
            ****************************************** *****************

P06278      DFKWHWYHFDGTDWDESRKLNRIYKFQGKAWDWEVSNENGNYDYLMYADIDYDHPDVAAE  240
104.K       DFKWHWYHFDGTDWDESRKLNRIYKFQGKAWDWEVSNENGNYDYLMYADIDYDHPDVAAE  240
            ************************************************************

P06278      IKRWGTWYANELQLDGFRLDAVKHIKFSFLRDWVNHVREKTGKEMFTVAEYWQNDLGALE  300
104.K       IKRWGTWYANELQLDGFRLDAVKHIKFSFLRDWVNHVREKTGKEMFTVAEYWQNDLGALE  300
            ************************************************************

P06278      NYLNKTNFNHSVFDVPLHYQFHAASTQGGGYDMRKLLNSTVVSKHPLKAVTFVDNHDTQP  360
104.K       NYLNKTNFNHSVFDVPLHYQFHAASTQGGGYDMRKLLNGTVVSKHPLKSVTFVDNHDTQP  360
            **************************************.*********:***********

P06278      GQSLESTVQTWFKPLAYAFILTRESGYPQVFYGDMYGTKGDSQREIPALKHKIEPILKAR  420
104.K       GQSLESTVQTWFKPLAYAFILTRESGYPQVFYGDMYGTKGDSQREIPALKHKIEPILKAR  420
            ************************************************************

P06278      KQYAYGAQHDYFDHHDIVGWTREGDSSVANSGLAALITDGPGGAKRMYVGRQNAGETWHD  480
104.K       KQYAYGAQHDYFDHHDIVGWTREGDSSVANSGLAALITDGPGGAKRMYVGRQNAGETWHD  480
            ************************************************************

P06278      ITGNRSEPVVINSEGWGEFHVNGGSVSIYVQR                              512
104.K       ITGNRSEPVVINSEGWGEFHVNGGSVSIYVQR                              512
```

Figure S3. Sequence alignment of the thermostable α-amylase (Amylase-104.K) from *B. licheniformis* 104.K and the UniProt-annotated *B. licheniformis* α-amylase sequence (UniProt ID: P06278). Amino acid variation positions are shown in bold and highlighted in green. The alignment was generated using Clustal Omega.

```
ACN88151.1      MKQQKRLYARLLTLLFALIFLLPHSAAAAANLNGTLMQYFEWYMPNDGQHWKRLQNDSAY   60
104.K           MKQQKRLYARLLTLLFALIFLLPHSAAAAANLNGTLMQYFEWYMPNDGQHWKRLQNDSAY   60
                ************************************************************

                                                             107
ACN88151.1      LAEHGITAVWIPPAYKGTSQADVGYGAYDLYDLGEFHQKGTVRTKYSTKGELQSAIKSLH   120
104.K           LAEHGITAVWIPPAYKGTSQADVGYGAYDLYDLGEFHQKGTVRTKYGTKGELQSAIKSLH   120
                **********************************************.*************

                                                         163
ACN88151.1      SRDINVYGDVVINHKGGADATEDVTAVEVDPADRNRVISGEHRIKAWTHFHFPGRGSTYS   180
104.K           SRDINVYGDVVINHKGGADATEDVTAVEVDPADRNRVISGEHLIKAWTHFHFPGRGSTYS   180
                ****************************************** *****************

ACN88151.1      DFKWHWYHFDGTDWDESRKLNRIYKFQGKAWDWEVSNENGNYDYLMYADIDYDHPDVAAE   240
104.K           DFKWHWYHFDGTDWDESRKLNRIYKFQGKAWDWEVSNENGNYDYLMYADIDYDHPDVAAE   240
                ************************************************************

ACN88151.1      IKRWGTWYANELQLDGFRLDAVKHIKFSFLRDWVNHVREKTGKEMFTVAEYWQNDLGALE   300
104.K           IKRWGTWYANELQLDGFRLDAVKHIKFSFLRDWVNHVREKTGKEMFTVAEYWQNDLGALE   300
                ************************************************************

                                                               349
ACN88151.1      NYLNKTNFNHSVFDVPLHYQFHAASTQGGGYDMRKLLNGTVVSKHPLKAVTFVDNHDTQP   360
104.K           NYLNKTNFNHSVFDVPLHYQFHAASTQGGGYDMRKLLNGTVVSKHPLKSVTFVDNHDTQP   360
                ************************************************:***********

ACN88151.1      GQSLESTVQTWFKPLAYAFILTRESGYPQVFYGDMYGTKGDSQREIPALKHKIEPILKAR   420
104.K           GQSLESTVQTWFKPLAYAFILTRESGYPQVFYGDMYGTKGDSQREIPALKHKIEPILKAR   420
                ************************************************************

ACN88151.1      KQYAYGAQHDYFDHHDIVGWTREGDSSVANSGLAALITDGPGGAKRMYVGRQNAGETWHD   480
104.K           KQYAYGAQHDYFDHHDIVGWTREGDSSVANSGLAALITDGPGGAKRMYVGRQNAGETWHD   480
                ************************************************************

ACN88151.1      ITGNRSEPVVINSEGWGEFHVNGGSVSIYVQR       512
104.K           ITGNRSEPVVINSEGWGEFHVNGGSVSIYVQR       512
                ********************************
```

Figure S4. Sequence alignment of the thermostable α-amylase (Amylase-104.K) from *B. licheniformis* 104.K and a synthetic α-amylase construct (GenBank ID: ACN88151.1). Amino acid variation positions are shown in bold and highlighted in cyan. The alignment was generated using Clustal Omega.
